# Assessing growth in children and adolescents with Avoidant/Restrictive Food Intake Disorder

**DOI:** 10.1186/s40337-024-01034-8

**Published:** 2024-06-14

**Authors:** Anna B. Tanner, Tracy K. Richmond

**Affiliations:** 1 Department of Pediatrics, Emory University, Atlanta, GA 30322 USA; 2 Division of Adolescent/Young Adult Medicine, Boston Children’s Hospital, Boston, MA 02115 USA; 3 Department of Pediatrics, Harvard Medical School, Boston, MA 02115 USA

**Keywords:** Eating disorders, Growth and development, ARFID

## Abstract

**Background:**

Although growth delays and disruption are a well described medical complication of restrictive eating disorders in children and young adolescents, this complication has received less attention in patients with Avoidant/Restrictive Food Intake Disorder (ARFID). Patients with ARFID have challenges with adequacy of food volume and variety that are not related to body image but are instead related to lack of interest in eating, sensory concerns, and/or fears of aversive consequences. Because onset of ARFID is commonly before puberty, concerns regarding growth adequacy may present an additional treatment challenge and a unique opportunity for support.

**Review:**

Child and adolescent patients with other restrictive eating disorders are at risk of irreversible deleterious impact on growth and development, particularly when onset is before or around puberty. Although faltering growth is a defining feature of ARFID, less attention has been paid to methods for examining growth concerns in young patients with ARFID and training providers to assess growth adequacy when prepubertal and peripubertal patients present with this diagnosis. Providers working with patients under 18 years of age with eating disorders will benefit from the tools discussed in this narrative review to adequately assess growth and development against genetic potential, recognize alterations in growth that are a result of nutritional deficiencies, and support and maximize catch-up growth and development when it has been impaired.

**Conclusion:**

Established pediatric growth monitoring tools and techniques to assess adequacy of growth can be applied to child and adolescent patients presenting with ARFID. These tools can improve long term outcomes in linear height for these patients and allow for monitoring during and after treatment until growth and development is complete. Medical providers caring for patients presenting with ARFID will need to establish best practices for assessing and monitoring growth.

## Abstract (plain language summary)

Providers caring for younger patients with eating disorders must remember that children should be growing, and adolescents must progress through puberty. Linear height gain, as well as bone and brain development, hinge upon adequate nutrition. Patients with Avoidant/Restrictive Food Intake Disorder (ARFID) are at risk for inadequate nutrition and will need an assessment of growth at presentation, as well as ongoing medical support to assure that growth is on track to achieve predicted adult height. Lack of attention to these developmental concerns in a critical window may allow growth to be lost. For medical providers, additional skills will be needed to assess young patients with ARFID and assist them in resuming and achieving growth that matches their genetic potential.

## Background

Addressing growth concerns in young patients with restrictive eating disorders has become an area of focused attention as the average age of onset has decreased and incidence has increased, especially during the post-COVID years [1]. The average age of onset of anorexia nervosa (AN), one of the best-described restrictive eating disorders, is 12 years [2–4], an age that typically falls during critical years for linear growth and pubertal development. This epidemiological shift has brought new urgency to the identification and management of growth concerns by medical providers caring for younger patients with restrictive eating disorders. Avoidant Restrictive Food Intake Disorder (ARFID) is a more recently described eating disorder characterized by restriction not motivated by body shape or size concerns. Patients with ARFID tend to have onset at an even younger age than those with AN (10.7 years compared to 12.7 for those with AN) [5–8], further increasing risk to growth and pubertal development. Linear height gain and pubertal progression, as well as bone and brain development, hinge upon nutritional adequacy and may all be adversely affected in children and young adolescents with ARFID [9–11] in potentially greater degree than has been recognized in those with AN. Despite this, no specific guidelines or recommendations have been developed to guide medical providers on assessing children and young adolescents with ARFID for growth concerns.

ARFID was only recently codified in the fifth version of the Diagnostic and Statistical Manual of Mental Disorders (DSM-5), published in 2013 [12]. ARFID is now recognized as having multiple subtypes—fear of aversive consequences, low interest in food, and sensory sensitivity—that may be overlapping. Thus, patients with ARFID can present in diverse ways, often leading to delay in identification. Despite the heterogeneity, faltering growth has been identified as a defining feature of ARFID [13] with pubertal delay and growth retardation established as medical complications of ARFID [14–16]. Thus, all prepubertal and peripubertal patients with ARFID require astute attention to growth adequacy. However, the heterogeneity of presentations may mask growth concerns and may preclude the adoption of a single approach to assessing growth in this population. Medical providers must remember that patients with all three ARFID subtypes are at risk for growth concerns. These concerns include slow weight gain, slow height gain, overt growth stunting, and/or growth that does not meet genetic potential.

Providers following the current Society for Adolescent Health and Medicine (SAHM) guidelines for the medical assessment of youth with restrictive eating disorders may focus on identifying children and adolescents in need of acute medical stabilization due to physiologic instability or laboratory or electrocardiogram abnormalities [17]. When providers focus solely on vital signs, laboratory studies and tests, they may fail to identify patients with ARFID who are also in urgent need of greater intervention. In one study, patients with ARFID with lack of appetite were much more likely to have faltering growth (68%) than bradycardia (1%) [16]. The physiologic changes identified in the SAHM guidelines are most commonly seen in patients with abrupt weight loss and may be more common in patients with AN than in patients with ARFID. Arrested growth and development however, remain a supporting factor for the consideration of hospitalization in the SAHM guidelines and must be evaluated during the initial medical assessment and then followed closely in all patients in outpatient care.

Prior recommendations for the pediatric medical evaluation of patients presenting with ARFID have called for an evaluation of vital signs and physical symptoms, micronutrient deficiencies, weight loss, pubertal assessment and an assessment for weight or height gain failure [15]. We offer guidance in assessing growth for providers caring for youth with ARFID using well-established growth assessment tools. We apply these growth assessment tools to examples of the currently described ARFID subtypes [18] to illustrate various presentations.

### The growth evaluation of child and adolescent patients with fear of aversive consequences subtype

Patients presenting with the ARFID subtype characterized by fear of aversive consequences often present with an acute change in eating patterns, frequently following a frightening event (e.g., choking on a food item). In one study at a tertiary care program, the most common presentations were a fear of vomiting (50%) and a fear of choking (23%), with significant enough direct medical complications to require acute medical hospitalization in 57% of patients [19]. Similar to patients with AN, these children and adolescents often have normal patterns of eating prior to illness onset with developmentally normal gains in weight and linear height. Parental observations and patient report of change in oral intake related to a fearful event often correlate with changes that can be observed on growth charts.

The initial medical evaluation of all children and young adolescents should include an objective assessment of growth by a review of growth charts. Children who are well nourished grow at predictable rates and track along height, weight, and Body Mass Index (BMI) percentile curves [20]. In the United States, World Health Organization (WHO) growth charts are used until age 2 years (https://www.who.int/tools/child-growth-standards/standards), and then Centers for Disease Control and Prevention (CDC) growth charts are used from ages 2 to 20 years (https://www.cdc.gov/growthcharts/charts.htm). In other countries, use of the WHO growth charts may continue beyond age 2 years old.

In children and adolescents without medical or nutritional concerns, downward shifts across 2 or more percentile curves (e.g., from the 75th to the 25th) after age 3 or 4 are uncommon. When children are malnourished, they may stop gaining weight at a predictable rate or altogether, and then will stop making gains in linear height. On review of patterns on growth charts, this will be represented as a downward shift in percentiles first on the weight growth chart and subsequently on the height growth chart [20]. Weight gain stagnation and slowing or cessation of linear growth may be difficult to detect without astute growth chart analysis. These deviations from prior growth may be less obvious if growth is not plotted on an established growth curve, thus providers are encouraged to review growth on growth curves such as those from the CDC. For patients with decelerated linear height gains due to inadequate oral intake, the primary goal of treatment should be to support nutritional rehabilitation to restore and maintain normal growth.

**Fig. 1:**
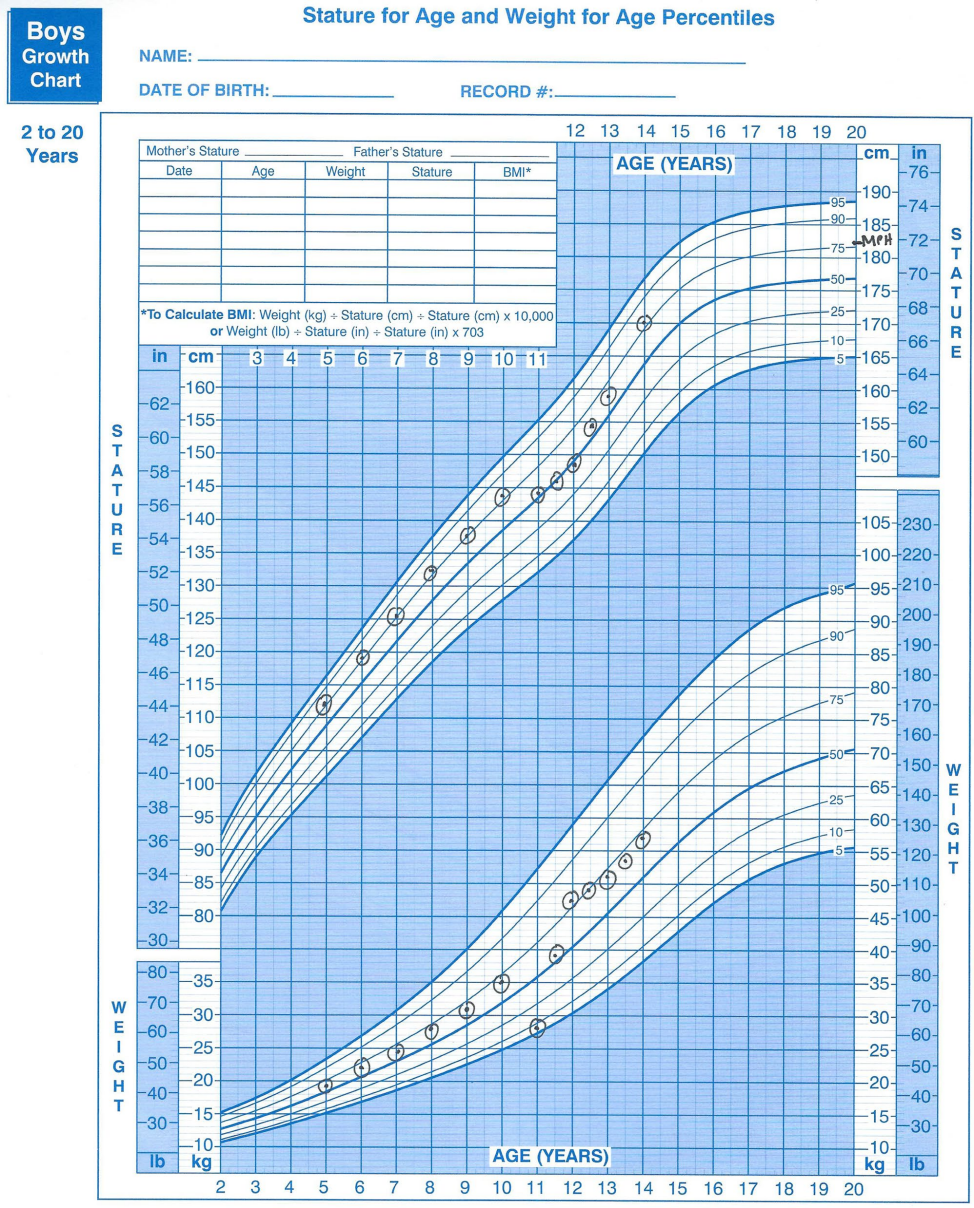
Example growth chart of patient with ARFID fear of aversive consequences subtype

Unlike patients with other ARFID subtypes, patients with fear of aversive consequences subtype typically have an abrupt change in weight that can be easily noted on the weight growth chart. Additionally, if considerable time lapses prior to presentation, deceleration of height across percentiles will also be noted. Figure 1 shows a hypothetical example of a typical growth chart based on our experience with patients with a fear of aversive consequences. Note acute weight loss with weight crossing percentile lines, slowing of linear height gains such that height crosses percentile lines, and resumption of weight gain prior to resumption of linear height to previous appropriate weight and height curves.

One additional consideration for these patients will be the duration of time patients need to receive intensive eating and weight-focused care. We often describe 2 phases in care: (1) Weight restoration and (2) Resumption/maintenance of developmentally appropriate weight and height gains. Once restoration to prior weight and height percentiles has been achieved, children and adolescents should continue to make gains in height and weight and progress through puberty until late adolescence. Weight goals should be set to anticipate the additional growth expected over the time frame that weight restoration may occur. For example, if a patient has a significant amount of weight to restore and it is anticipated to take up to 6 months to restore, the weight goal should be set at the expected weight for the age 6 months to the future. Medical providers should prepare patients and families that ongoing assessments of growth will continue until adult height is achieved and patients are weight stable with no other medical concerns at their final adult height. Note that for the sample patient in Fig. 1, even at age 14 years old, significant height and weight gain still need to occur. Ongoing monitoring of weight, height, and pubertal development, perhaps quarterly, would be prudent to establish that prior behaviors impacting growth do not return and that intake remains adequate for restored gains along previously established weight and height percentiles.

### The growth evaluation of child and adolescent patients with a lack of interest in eating subtype

In contrast to patients presenting with a fear of an aversive consequence, many patients seen for medical evaluation with lack of interest subtype of ARFID will describe behaviors that date back to early childhood or even infancy and toddlerhood. They or their caregivers will often describe an apparent lack of appetite resulting in only small volumes of food intake. Caregivers may describe “eating but not enough” and “not initiating eating” [16]. Despite, or perhaps because of, long-standing concerns, these patients often present at older ages than patients with the other ARFID subtypes [21]. Caregiver input regarding the onset of lack of interest behaviors helps in the accurate interpretation of growth charts.

Timing of onset of lack of interest eating behaviors can impact both the duration and severity of findings at time of presentation. Parents who describe an infant that would not stay at the breast may have a child presenting for care with inadequate weight gain dating back to infancy. When lack of interest in eating presents in the toddler years, caregivers may describe a child easily distracted at the table, who took a long time to consume food and required extra effort for intake at meals. These patients may have inadequate weight gain dating back to the toddler years. Young children with a lack of interest in eating may have behaviors that include being called to the table multiple times for meals, standing at meals, eating a few bites before asking to be excused, and missing meals or snacks if not prompted to eat. Again, changes in rate of weight gain may be seen at the noted onset of these behaviors. As patients enter school, meals away from caregivers, at school, camps, and friends’ houses may be missed without prompts and support from regular caregivers. Because these behaviors start so early and thus become normalized, patients may not seek care until late in high school or even at college age. Yet changes in growth may be noted with astute attention to growth charts during the initial medical history.

Of note, with the increased availability of over-the-counter oral supplements or meal replacements (e.g., Boost or Ensure), many of these concerns may be overlooked, minimized, or dismissed as the use of such supplements may mask the underlying concerns. Medical providers must listen carefully to caregivers. Caregivers who are providing regular oral supplementation due to concerns over poor oral intake should not be disregarded. Reliance on supplementation is a defining feature of ARFID in the DSM-5 yet may be missed as a concern and may mask overt changes on the growth curve.

**Fig. 2:**
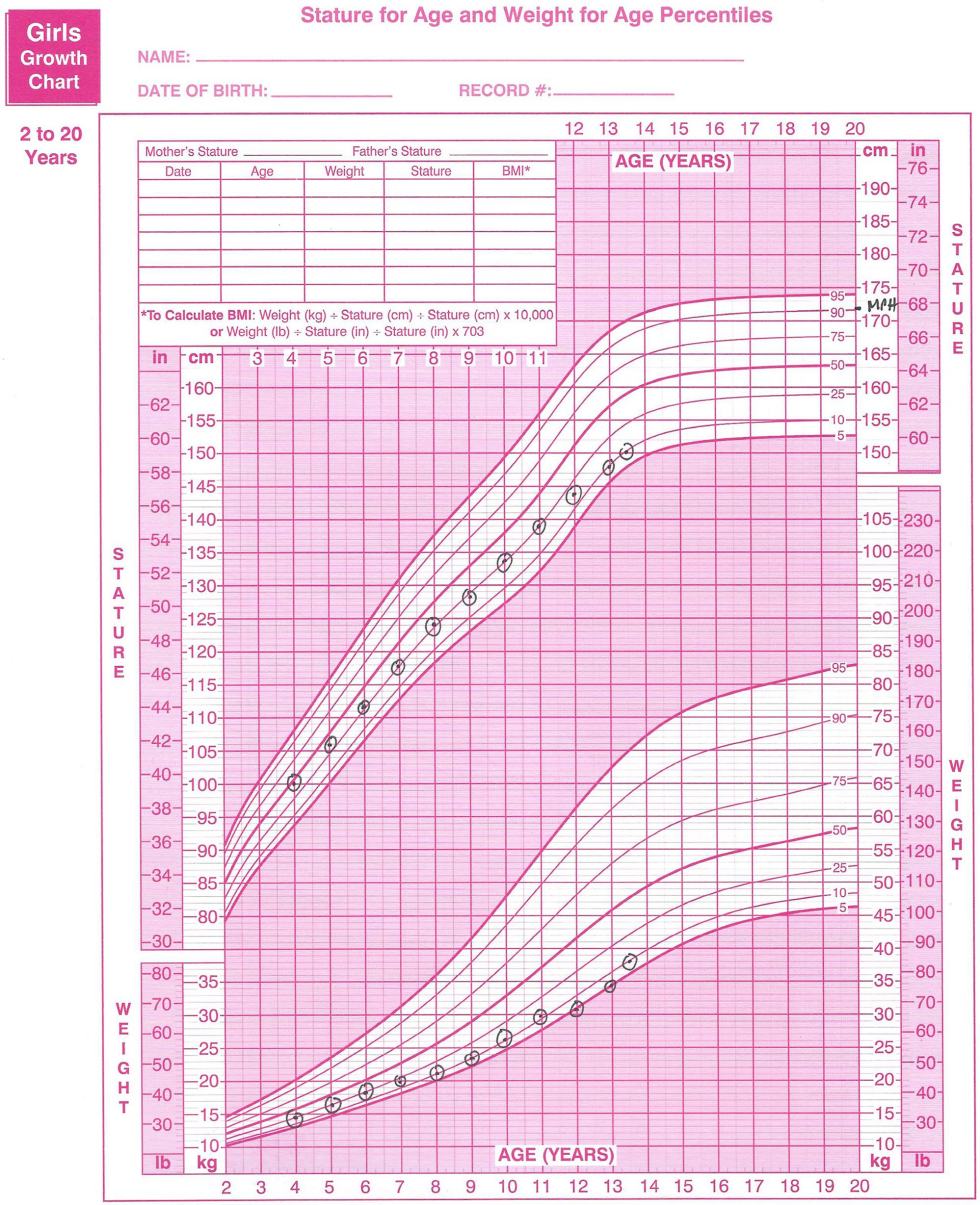
Example growth chart of a patient with ARFID lack of interest in eating subtype

Additional challenges for these patients have been noted to occur at times of increased energy needs such as during the pubertal growth spurt [16] or the addition of an intensive sport. Figure 2 demonstrates a hypothetical example based on our clinical experience of a child with slow but steady growth due to caregiver support and the use of supplements/meal replacements. However, this parental support was inadequate to meet increased energy needs during puberty. These unmet energy needs for pubertal development outpaced the child’s intake and resulted in slowed weight gain with a resultant deceleration of height gain. As a result, the patient had a downward shift of both weight and height percentiles.

For medical providers accustomed to caring for children and young adolescents who had normal growth and development prior to illness onset, there are tools to help understand each child’s growth potential. A pediatric tool that can be useful in the assessment of growth rate adequacy in a child with long standing volume issues is the mid-parental height (MPH) calculation. Calculation of the MPH helps to understand the child’s likely growth potential based on parental patterns and genetics. Because patients with ARFID may have growth and pubertal delays, this may be dismissed as constitutional growth delay. However, these patients do not have additional late growth and thus, do not catch up. Clues that this may not be constitutional growth delay are normal parental pubertal timing and normal parental growth timing.

Providers can determine MPH with the following formula: [20]

- For biologic females: [(Father’s height (in) – 5) + Mother’s height (in)] / 2
- For biologic males: [Father’s height (in) + (Mother’s height (in) + 5)] / 2
- Predicted range: +/- 3 inches

Once calculated, the MPH can then be plotted on the patient’s growth chart. For patients with low volume intake due to ARFID, the observed height percentile may be lower than the height percentile of the MPH. This would indicate inadequate energy intake resulting in failure to meet genetic growth potential. In Fig. 2, observers will note that this patient had height growth along the 10th percentile but an expected MPH at the 90th percentile. Growth from the earliest recorded data, at age 4 years old, was not at the expected height percentile, indicating issues of undernourishing dating back to infancy or toddlerhood. For any patient with poor linear growth, nutritional rehabilitation to drive linear height gains while growth plates remain open is essential. Oral nutritional formula supplementation may be used in these patients to increase dietary volume and maximize energy intake while establishing definitive care for ARFID [22]. Allowing young patients to remain stalled in linear growth results in an unnecessary irreversible medical complication.

### The growth evaluation of child and adolescent patients with sensory sensitivity subtype

Child and adolescent patients with ARFID sensory sensitivity subtype typically present with a limited variety of accepted foods. They or their caregivers may report that they avoid certain foods due to taste, texture, smell, appearance, or temperature, or that they have difficulty digesting certain foods. These patients may present with reports that they only have a few foods that they can consume and that they can only eat preferred foods under specific conditions. These limitations on variety may lead to inadequate volume, despite an espoused desire by patients to eat more and/or gain weight. These patients may feel hunger and are often willing to eat the same foods and same brands, meal after meal and day after day, in order to take in an adequate volume of food.

The elimination of specific foods from the diet of a child is known to potentially result in growth restriction and nutritional deficits and is thus advised with caution in fields such as pediatric allergy [23]. Patients with sensory sensitivity subtype ARFID often electively and persistently omit more than one food, or an entire class of foods, and thus are at risk for growth delays. Lack of variety may cause these patients to have persistent or intermittent issues with adequate caloric consumption, in addition to the nutritional issues that result directly from limited variety. Volume challenges can lead to growth that lags genetic potential, much like that seen in patients with lack of interest in eating. Additional challenges to consuming adequate volume include patients tiring of an accepted food and texture issues that impair use of supplements.

Struggles to consume adequate volume may increase as these patients age and pass the window of acceptable picky eating. Estimates vary on the prevalence of childhood selective eating; however, rates may be as high as 50% [24] and this behavior typically improves by early or middle childhood without treatment and no adverse impact on growth. In patients with ARFID and sensory sensitivity, these issues do not improve. Families often take extensive measures to ensure that accepted foods are provided in adequate volumes and with adequate frequency.

An additional concern in this profile group, are comorbid issues, such as gastrointestinal concerns. In one study, over 80% of patients with ARFID had complaints of gastrointestinal symptoms, and these concerns were associated with slower weight gain [25]. In patients who require increased oral intake, finding adequate foods in enough volume to restore normal growth and development can be particularly challenging.

For patients with ARFID sensory sensitivity subtype, volume and variety inadequacy can result in poor weight gain. Even with intermittent or consistent supplement use, total calories consumed may be inadequate to support normal growth and development. If weight gain is steady and patients remain in the same weight and height percentiles, medical providers may not be concerned, even when parental concerns persist. Unless detailed analysis of linear growth is completed, these concerns may remain undetected and thus unresolved.

**Fig. 3:**
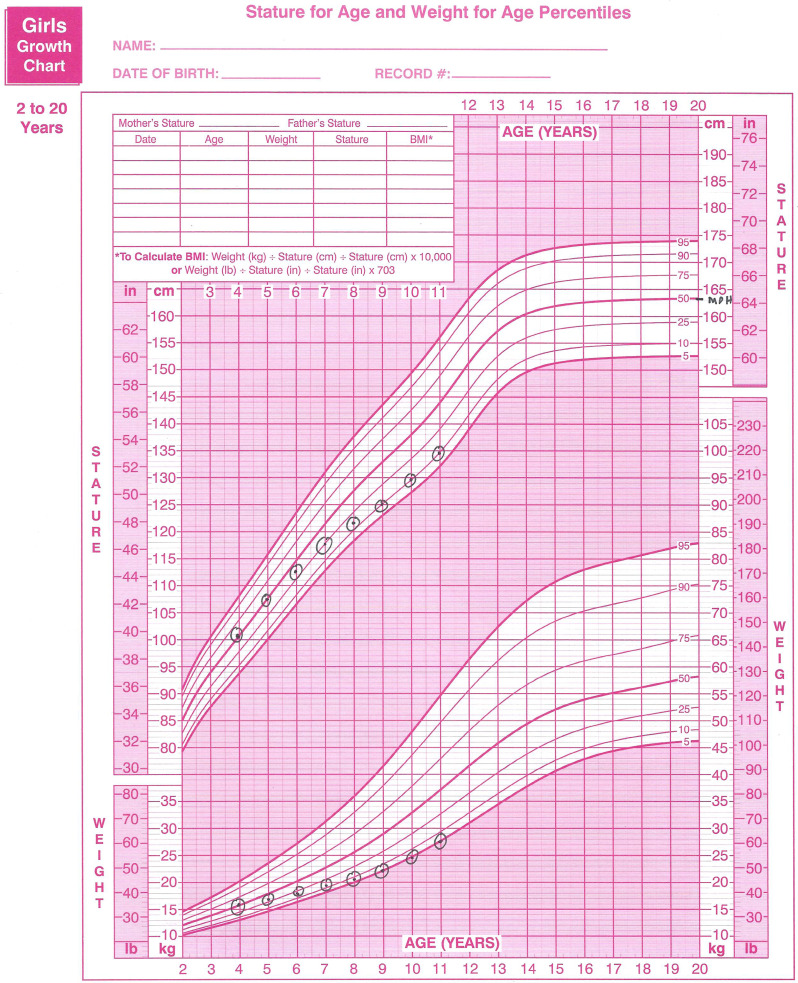
Example growth chart of a patient with ARFID sensory sensitivity subtype

The timing of the worsening of these challenges may be apparent as slowing on the growth curve, as illustrated for a hypothetical patient in Fig. 3. This patient was at the 50th percentile for height at age 4 years old, which corresponded to her predicted MPH percentile. Struggles to consume adequate variety led to struggles to consume adequate volume. Weight gain slowed, followed by slowed height gain. As children with sensory sensitivity spend more time away from parents, attending school, camp, or working, they may have times without meal support resulting in increased caloric intake inadequacy. Restoring normal growth patterns is essential.

### Evaluation of child and adolescent patients with more than one ARFID subtype

Providers should remember that the ARFID profiles are not exclusive, and thus patients may present with overlapping features. A recent study described common combination patterns of fear + low appetite and fear + sensory sensitivity + low appetite [21]. Patients with overlapping features may present like one case reported of an 11-year-old female with a history of selective eating since infancy, crying with initial solid food exposure, coaxing from parents to complete meals, and strict food preferences. This patient did not present for an eating-focused evaluation until she had difficulty eating solid food following an episode of choking [26]. Additional studies are needed to establish the prevalence of these combined presentations in other clinical settings. In these patients, an even more detailed history and growth evaluation may be needed to detect how ARFID behaviors impacted weight gain and linear height.

### Critical factors in monitoring and supporting growth in all ARFID subtypes

A critical step for all children and adolescents presenting to medical care for any ARFID subtype is to assess and determine an expected weight and height. Our treatment approach emphasizes providing adequate caloric volume over variety in the initial stage to weight restore and begin to restore linear height. Once the pace of normal growth has been restored, patients with ARFID will need close and ongoing medical supervision to ensure adequacy of weight gain to continue to drive height. Patients with time and energy for the pubertal growth spurt, may have greatly increased energy needs during this time and may require additional support and more frequent monitoring. Although monitoring weight is a key feature of all treatments for patients with eating disorders, for children and adolescents with ARFID, monitoring linear growth may be a better predictor of illness and more important for long term outcomes.

### Key points for medical providers assessing growth in patients in ARFID

1. All children and adolescents with ARFID should be assessed for linear growth concerns.
2. Linear height and growth in children and adolescents with ARFID should be compared to genetic potential.
3. Growth charts are critical tools for assessing height and weight patterns and changes in child and adolescent patients with ARFID.
4. Calculation of the MPH can be an additional tool in the evaluation of growth adequacy for children and adolescents with ARFID.
5. The initial treatment goal for children and adolescent with ARFID should be ensuring adequate weight gain to restore established or genetically predicted linear height percentiles.
6. When caring for adolescent patients with ARIFD, providers must remember that delayed puberty can be a manifestation of nutritional inadequacy and may not be reassuring that patients will have additional time to grow.
7. Patients with ARFID who are underweight or have growth concerns may need to work on volume adequacy before variety.
8. Patients with ARFID may need to use supplements as a bridge to quickly restore caloric adequacy until definitive treatment can be established.
9. Weight targets cannot be static in patients with ARFID with growth potential but should be adjusted with age to ensure linear growth continues.
10. Monitoring height may be of greater importance than monitoring weight in young patients with ARFID.
11. Time is of the essence for linear growth. Help patients with ARFID and growth concerns get back on track as quickly as possible.

## Conclusion

Although faltering growth is a defining feature of ARFID, little attention has been paid to crafting specific medical recommendations to evaluate for growth stunting and remediate growth failure when present. Methodologies to monitor growth by monitoring linear height, have traditionally not been routinely incorporated into eating disorders care, but are inexpensive and indicate appropriate response to treatment for young patients. Child and adolescent patients with all three ARFID subtypes are not immune to growth concerns and every initial and follow-up medical visit should include an evaluation of growth until patients reach linear height maturity. Growth curves, linear height measurements, and calculation of mid-parental height are well-established tools that can empower providers and families to make informed treatment decisions and improve long term outcomes for patients with ARFID.

## Data Availability

No datasets were generated or analysed during the current study.

## Abbreviations

ARFID: Avoidant/Restrictive Food Intake Disorder
CDC: Centers for Disease Control and Prevention
DSM-5: Diagnostic and Statistical Manual of Mental Disorders, fifth edition
MPH: Mid-parental height
SAHM: Society for Adolescent Health and Medicine
WHO: World Health Organization
